# Supplementary material for: Evolutionary History and Functional Divergence of Hydroxycarboxylic Acid Receptors in Primates
Source: Genome Biol Evol. 2026 May 26;18(6):evag126. doi: 10.1093/gbe/evag126 (PMC13237431; doi:10.1093/gbe/evag126)
Supplement: evag126_Supplementary_Data [file evag126_Supplementary_Data.pdf]

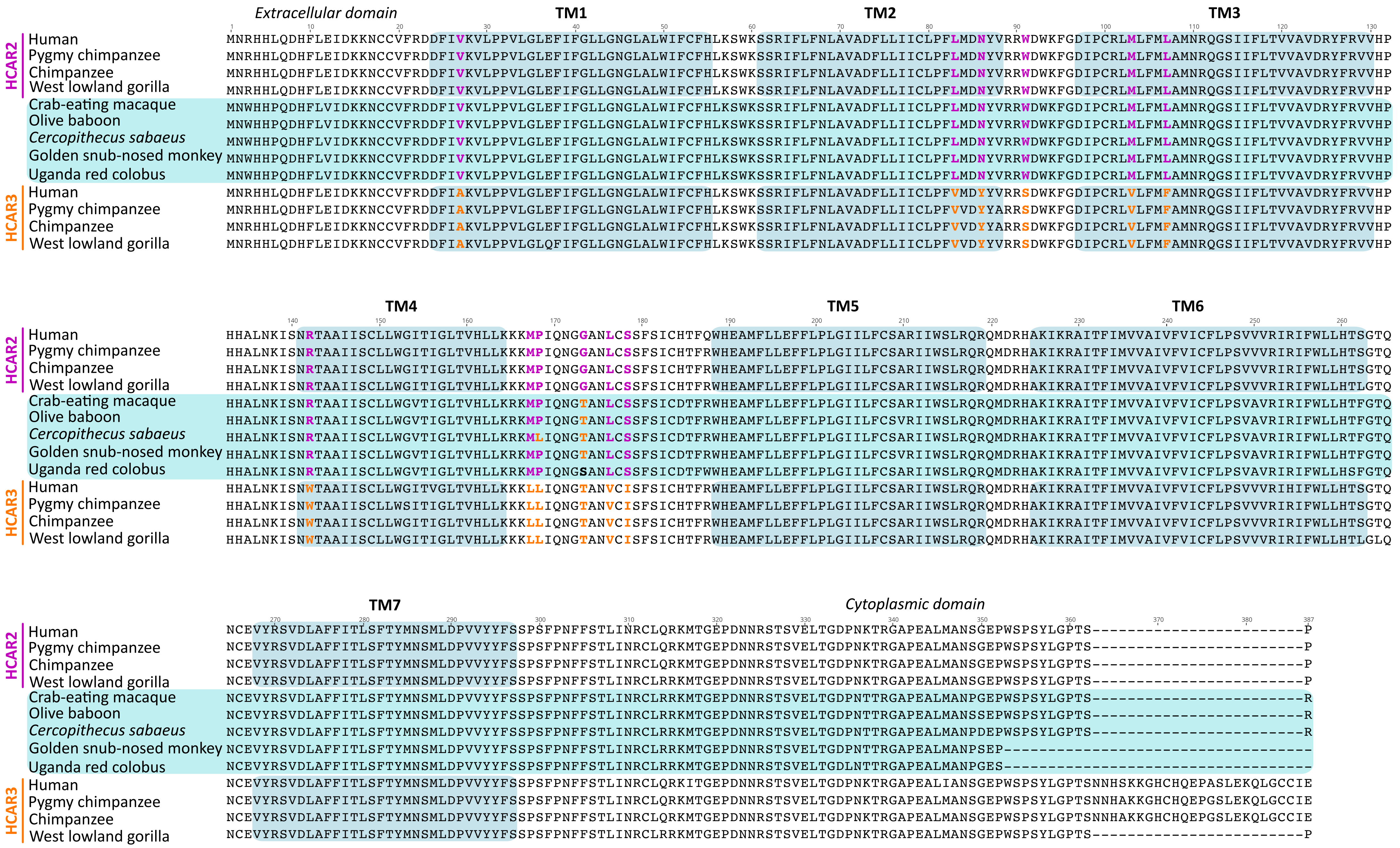

Supplementary Figure S1. Amino acid alignment of HCAR2 and HCAR3 sequences from human (*Homo sapiens*), chimpanzee (*Pan troglodytes*), pygmy chimpanzee (Pan paniscus), and western lowland gorilla (*Gorilla gorilla gorilla*), along with the single copy gene (HCAR2/3) from the crab-eating macaque (*Macaca fascicularis*), Olive baboon (*Papio anubis*), Cercopithecus sabaeus, Uganda red colobus (*Piliocolobus tephrosceles*), and golden snub-nosed monkey (*Rhinopithecus roxellana*). TM indicates transmembrane domains. Colored residues highlight the diagnostic alignment positions.

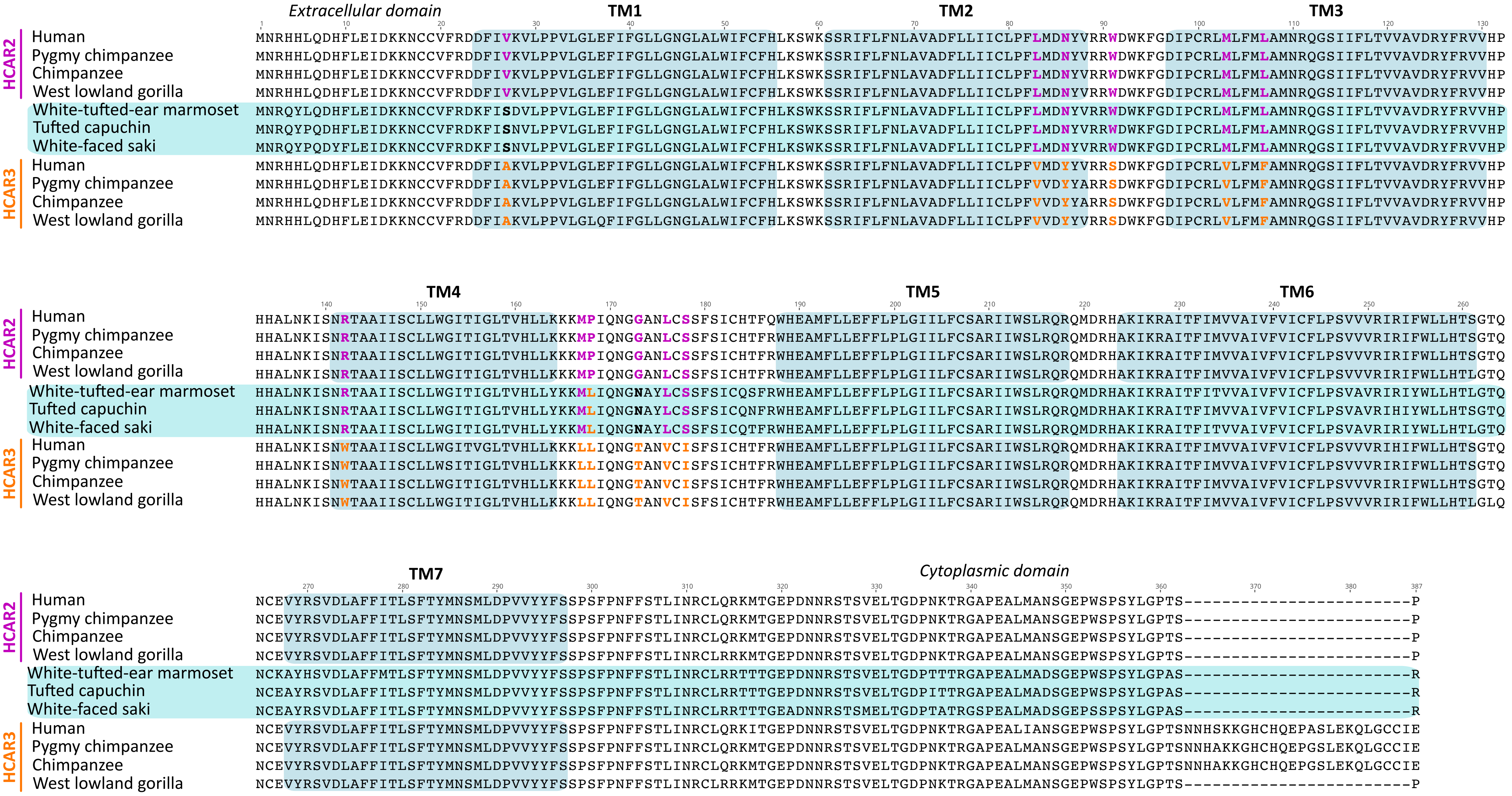

Supplementary Figure S2. Amino acid alignment of HCAR2 and HCAR3 sequences from human (*Homo sapiens*), chimpanzee (*Pan troglodytes*), pygmy chimpanzee (*Pan paniscus*), and western lowland gorilla (*Gorilla gorilla gorilla*), along with the single copy gene (HCAR2/3) from the white-tufted-ear marmoset (*Callithrix jacchus*), tufted capuchin (*Sapajus apella*), and white-faced saki (*Pithecia pithecia*). TM indicates transmembrane domains. Colored residues highlight the diagnostic alignment positions.

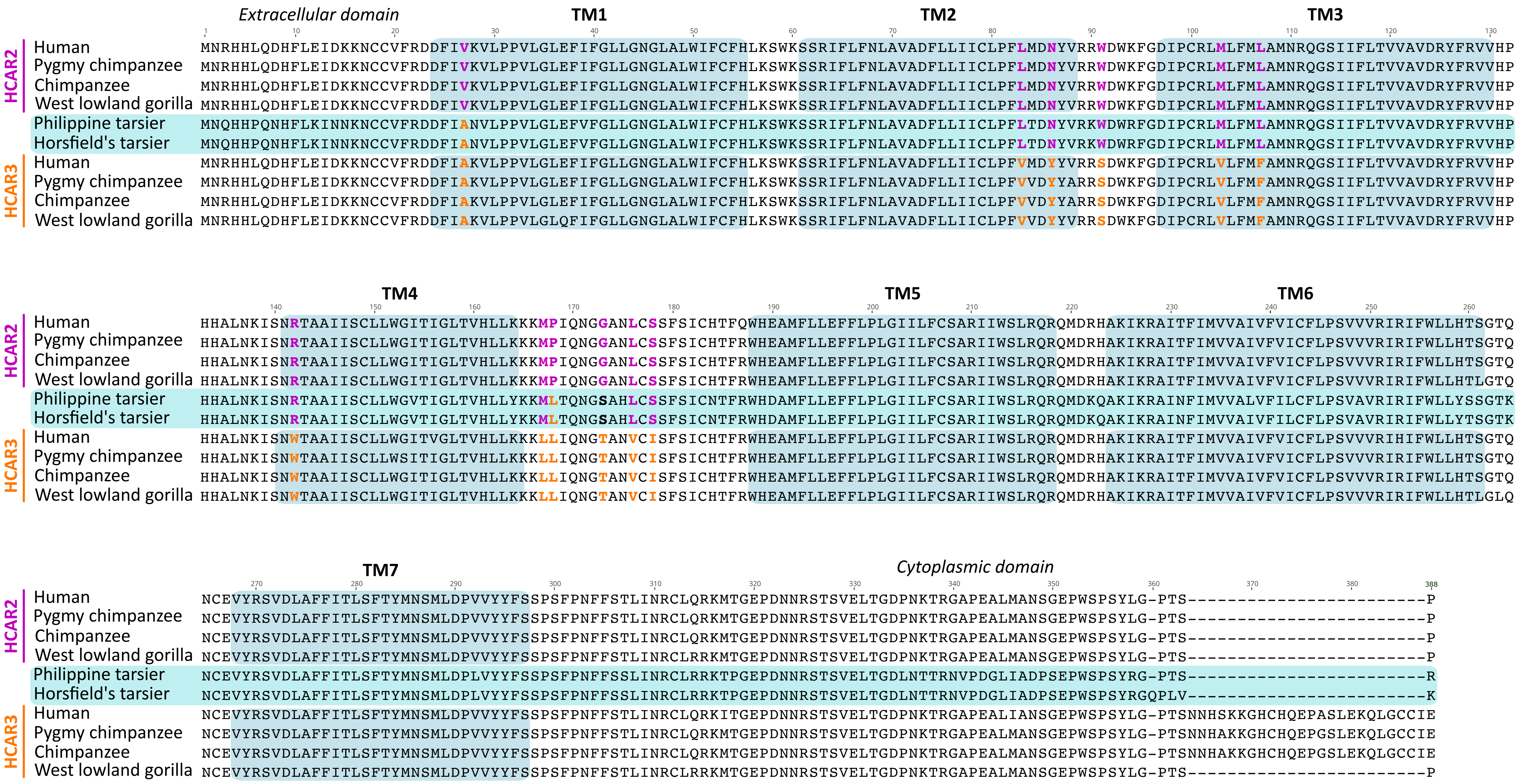

Supplementary Figure S3. Amino acid alignment of HCAR2 and HCAR3 sequences from human (*Homo sapiens*), chimpanzee (*Pan troglodytes*), pygmy chimpanzee (*Pan paniscus*), and western lowland gorilla (*Gorilla gorilla gorilla*), along with the single copy gene (HCAR2/3) from the Philippine tarsier (*Carlito syrichta*), and Horsfield's tarsier (*Cephalopachus bancanus*). TM indicates transmembrane domains. Colored residues highlight the diagnostic alignment positions.

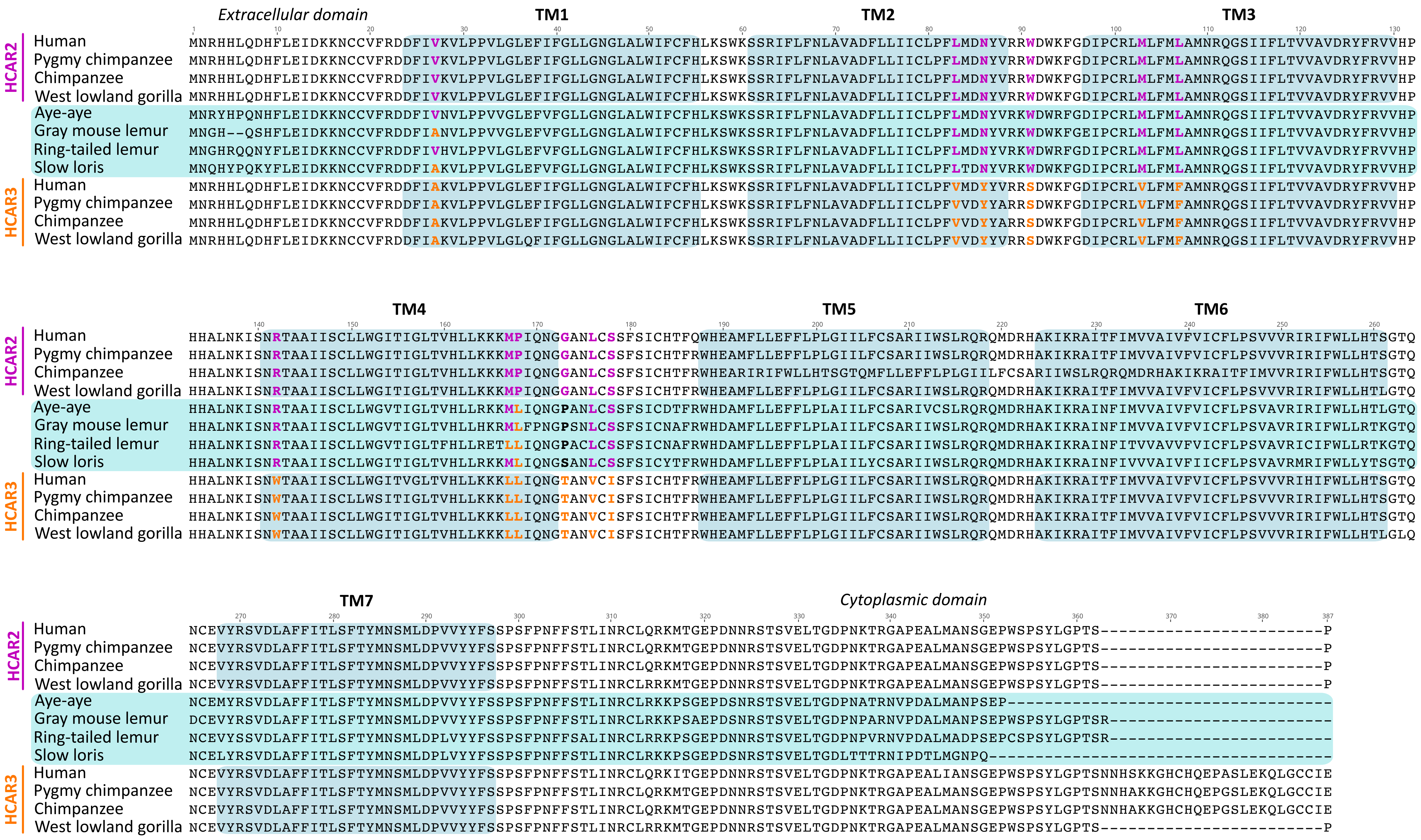

Supplementary Figure S4. Amino acid alignment of HCAR2 and HCAR3 sequences from human (*Homo sapiens*), chimpanzee (*Pan troglodytes*), pygmy chimpanzee (*Pan paniscus*), and western lowland gorilla (*Gorilla gorilla gorilla*), along with the single copy gene (HCAR2/3) from the aye-aye (*Daubentonia madagascariensis*), gray mouse lemur (*Microcebus murinus*), ring-tailed lemur (*Lemur catta*), and slow loris (*Nycticebus coucang*). TM indicates transmembrane domains. Colored residues highlight the diagnostic alignment positions.

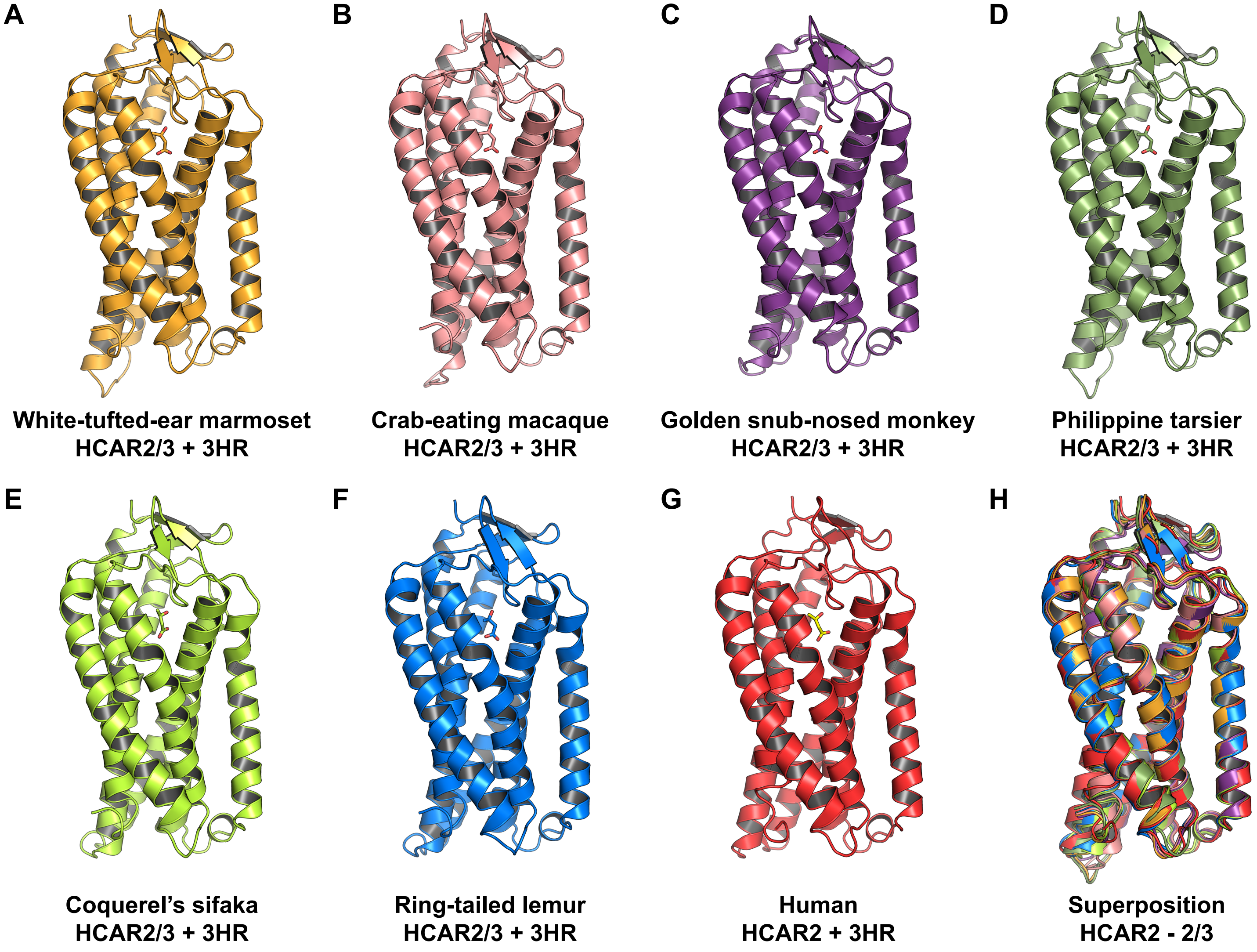

Supplementary Figure S5. Structural models of non-ape primate HCAR2/3 receptor bound to (D)-β-hydroxybutyrate generated by AlphaFold3. (A-F) Cartoon representations of HCAR2/3 receptors of White-tufted-ear marmoset (Callithrix jacchus) (A), Crab-eating macaque (Macaca fascicularis) (B), Golden snub-nosed monkey (Rhinopithecus roxellana) (C), Philippine tarsier (Carlito syrichta) (D), Coquerel's sifaka (Propithecus coquereli) (E), Ring-tailed lemur (Lemur catta) (F) bound to (D)-β-hydroxybutyrate (3HR), shown in stick representation. (G) For comparison, the cartoon representation of human HCAR2 is shown bound to 3HR, depicted in stick representation (PDB 8J6Q). (H) Cartoon representation of the C α coordinate superposition of the models shown in A to G that showed a RMSD of 0.801 ± 0.173 Å.

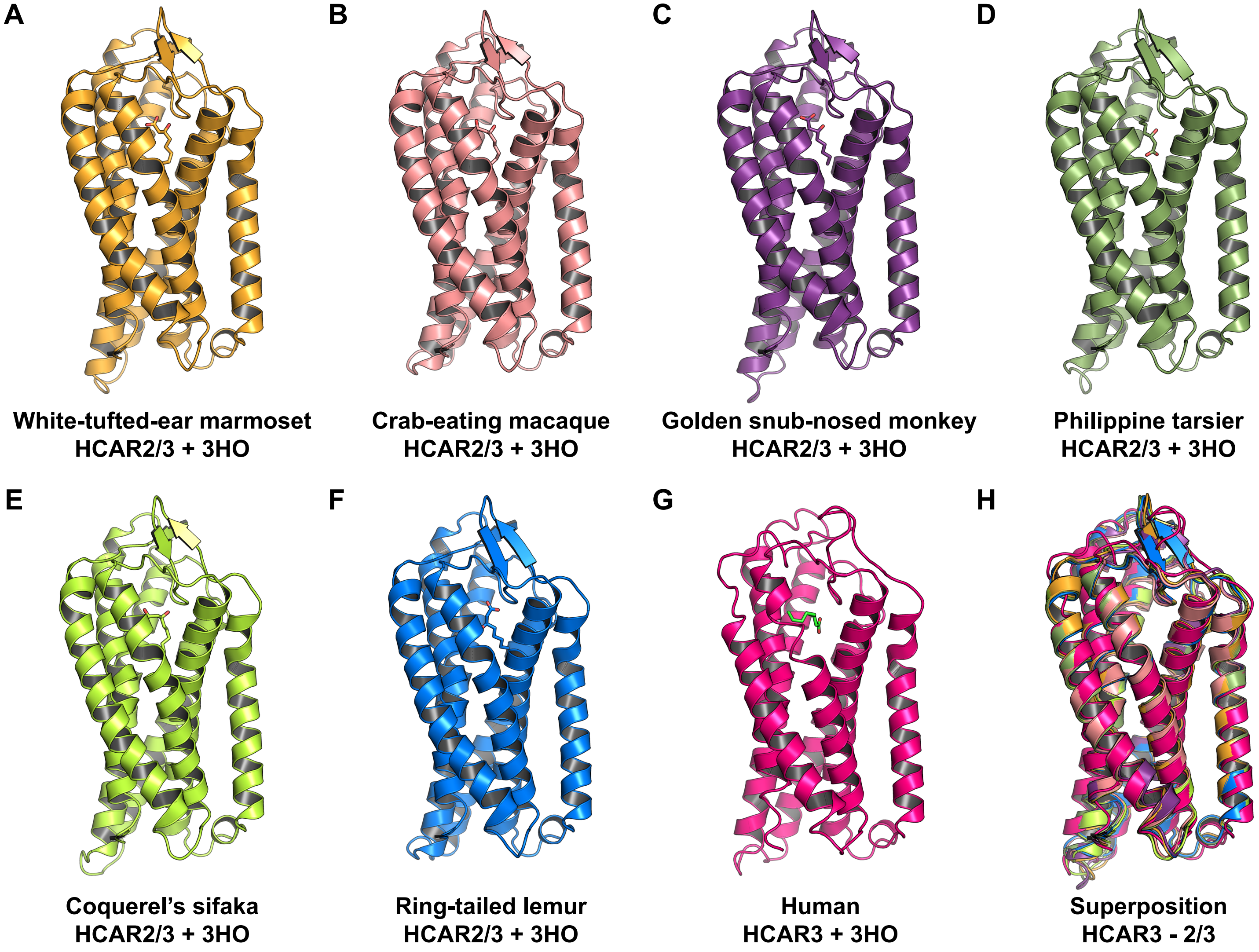

Supplementary Figure S6. Structural models of non-ape primate HCAR2/3 bound to 3-hydroxyoctanoate generated by AlphaFold3. (A-F) Cartoon representations of HCAR2/3 receptors of White-tufted-ear marmoset (Callithrix jacchus) (A), Crab-eating macaque (Macaca fascicularis) (B), Golden snub-nosed monkey (Rhinopithecus roxellana) (C), Philippine tarsier (Carlito syrichta) (D), Coquerel's sifaka (Propithecus coquereli) (E), Ring-tailed lemur (Lemur catta) (F) bound to 3-hydroxyoctanoate (3HO), shown in stick representation. (G) For comparison, the cartoon representation of human HCAR2 is shown bound to 3HO, depicted in stick representation (PDB 8JEF). (H) Cartoon representation of the Cα coordinates superposition of the models shown in A to G that showed a RMSD of 1.336 ± 0.082 Å.

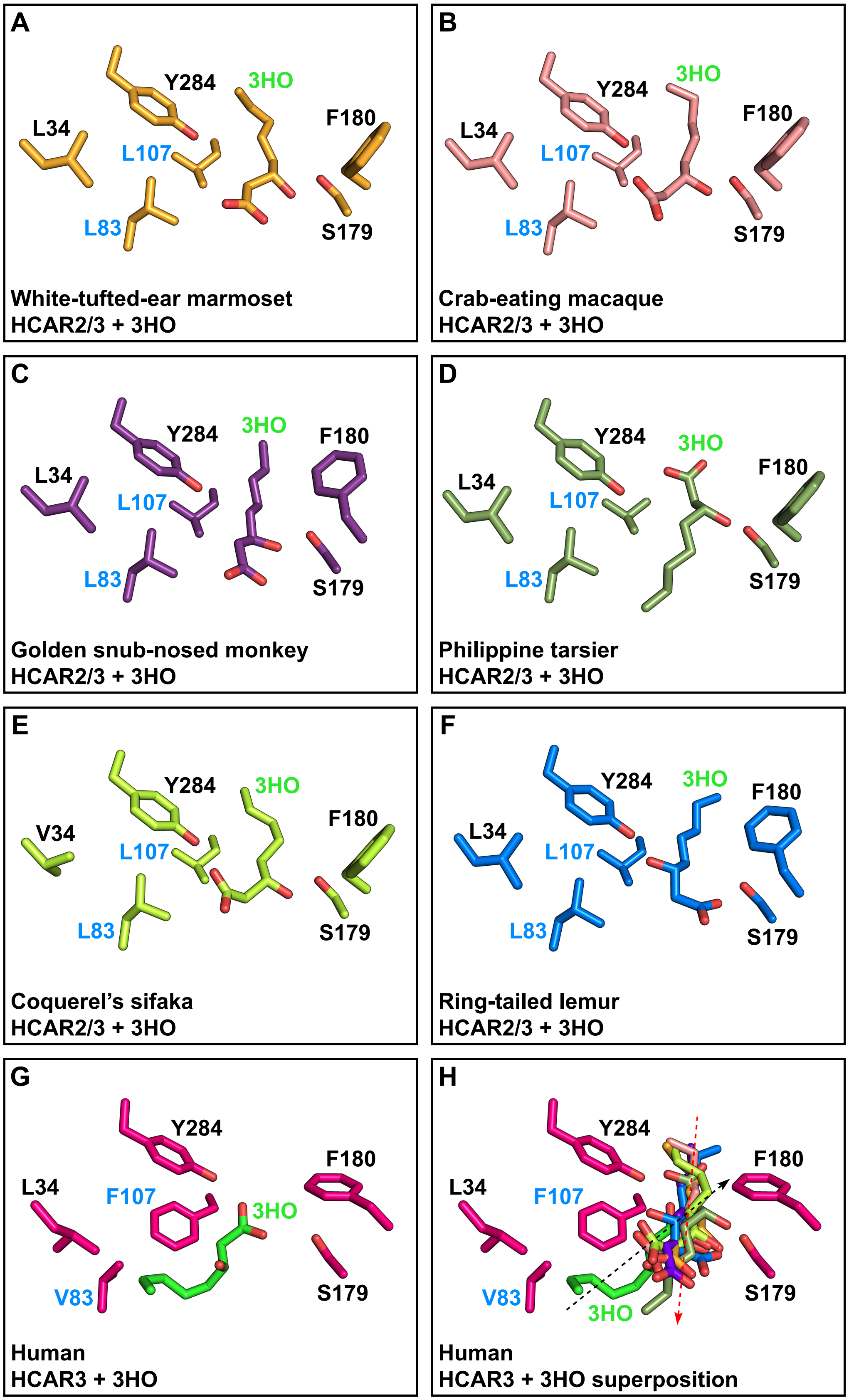

Supplementary Figure S7. Comparison of the ligand-binding pocket of HCAR2/3 models bound to 3-hydroxyoctanoate. (A-F) Stick representation of HCAR2/3 ligand binding pocket amino acid residues of the indicated non-ape primate species bound to 3-hydroxyoctanoate (3HO). (G) For comparison, human HCAR3 ligand-binding pocket amino acid residues are shown bound to 3HO (PDB 8JEF). (H) Comparison of the orientation of each 3HO shown in stick representation after the superposition of all HCAR2/3 models (A-F) with human HCAR3 (G). The dashed black arrow depicts the orientation of 3HO within the ligand-binding pocket of human HCAR3, and the dashed red arrow depicts the overall orientation of superposed 3HO within the ligand-binding pocket of each HCAR2/3 model, as shown in A to G. Distinguishing residues between HCAR2 and HCAR3 are highlighted in blue.

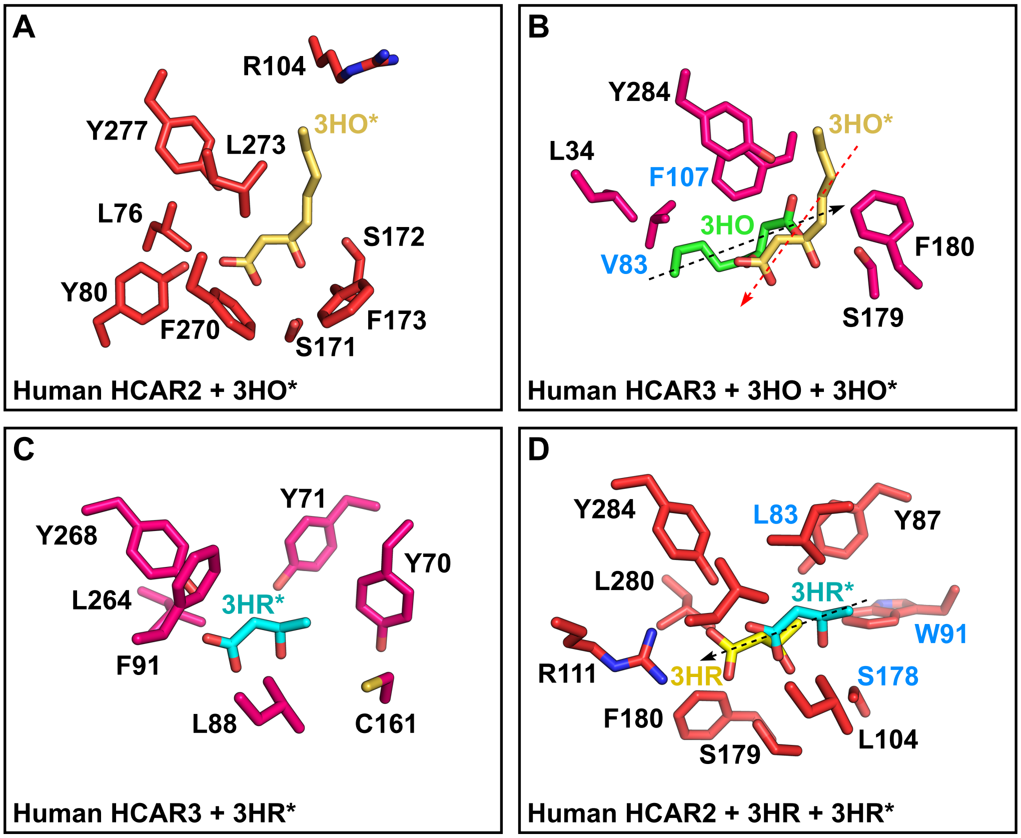

Supplementary Figure S8. Comparison of ligand swapping in human HCAR2 and HCAR3. (A) Stick representation of the human HCAR2 ligand-binding pocket, with amino acid residues shown in red, bound to the non-canonical ligand 3-hydroxyoctanoate (3HO*; carbon atoms in yellow), as predicted by AlphaFold3. (B) Stick representation showing the superposed 3HO* ligand from panel A within the ligand-binding pocket of human HCAR3 (residues shown in hot pink). The spatial orientation of the canonical ligand 3HO (green carbons) is compared with the superposed 3HO* (yellow carbons). The dashed black arrow indicates the orientation of 3HO, whereas the dashed red arrow indicates the opposite axial orientation adopted by 3HO*. (C) Stick representation of the human HCAR3 ligand-binding pocket (hot pink carbons) bound to the non-canonical ligand β-hydroxybutyrate (3HR*; cyan carbons), as predicted by AlphaFold3. (D) Stick representation showing the superposed 3HR* ligand from panel C within the ligand-binding pocket of human HCAR2 (residues shown in red). The spatial orientation of canonical 3HR (yellow carbons) is compared with that of superposed 3HR* (cyan carbons). The dashed black arrow depicts the parallel axial orientation of 3HR and 3HR*.

Supplementary Table S1. Accession numbers of the HCARs genes used in this study

Gene symbol	Scientific name	Common name	Lineage	Gene ID	Location
HCAR2	*Homo sapiens*	Human	Apes	ENSG00000182782	Chr12
HCAR2	*Pan troglodytes*	Chimpanzee	Apes	XM_024348111.3	Chr10
HCAR2	*Pan paniscus*	Pygmy chimpanzee	Apes	XM_034934721.3	Chr10
HCAR2	*Gorilla gorilla gorilla*	Western lowland gorilla	Apes	XM_004054070.5	Chr10
HCAR3	*Homo sapiens*	Human	Apes	ENSG00000255398	Chr12
HCAR3	*Pan paniscus*	Pygmy chimpanzee	Apes	XM_003812130.5	Chr10
HCAR3	*Pan troglodytes*	Chimpanzee	Apes	XM_009426485.5	Chr10
HCAR3	*Gorilla gorilla gorilla*	Western lowland gorilla	Apes	XM_031016622.3	Chr10
HCAR2$_H$	*Hoolock leuconedys*	Eastern hoolock gibbon	Apes		JBHMFN010000015:c90788023-90740669
HCAR3$_H$	*Hoolock leuconedys*	Eastern hoolock gibbon	Apes		JBHMFN010000015:c90788023-90740669
HCAR3$_H$	*Symphalangus syndactylus*	Siamang	Apes	XM_055239227.2	Chr13
HCAR2$_H$	*Hylobates agilis*	Agile gibbon	Apes		CAUYOT010050145:c4461-1
HCAR3$_H$	*Nomascus leucogenys*	Northern whitecheeked gibbon	Apes	XM_012510140.2	Chr10
HCAR2$_H$	*Symphalangus syndactylus*	Siamang	Apes	XM_055239226.2	Chr13
HCAR3$_O$	*Pongo abelii*	Sumatran orangutan	Apes	XM_003778149.5	Chr10
HCAR3$_O$	*Pongo pygmaeus*	Bornean orangutan	Apes	XM_054441852.1	Chr10
HCAR2$_O$	*Pongo abelii*	Sumatran orangutan	Apes	XM_024256763.2	Chr10
HCAR2$_O$	*Pongo pygmaeus*	Bornean orangutan	Apes	XM_054441851.2	Chr10
HCAR2/3	*Chlorocebus sabaeus*	Cercopithecus sabaeus	Old World monkey	XM_008005060.3	NW_23666037
HCAR2/3	*Papio anubis*	Olive baboon	Old World monkey	XM_003907318.5	Chr9
HCAR2/3	*Macaca fascicularis*	Crab-eating macaque	Old World monkey	XM_005596020.5	Chr11
HCAR2/3	*Rhinopithecus roxellana*	Golden snubnosed monkey	Old World monkey	XM_010368365.2	Chr10
HCAR2/3	*Piliocolobus tephrosceles*	Ugandan red Colobus	Old World monkey		PDMG03000004:120466402-120492050 (Chr10)
HCAR2/3	*Callithrix jacchus*	White-tufted-ear marmoset	New World monkey	XM_003733868.5	Chr9
HCAR2/3	*Sapajus apella*	Tufted capuchin	New World monkey	XM_032242811.1	NW_22436942
HCAR2/3	*Pithecia pithecia*	White-faced saki	New World monkey		JAPYXZ010000007:105145369-105158041 (Chr7)
HCAR2/3	*Carlito syrichta*	Philippine tarsier	Tarsiiformes	XM_008050397.1	NW_7089393
HCAR2/3	*Cephalopachus bancanus*	Horsfield's tarsier	Tarsiiformes		JAPVLO010000958:c264332-1
HCAR2/3	*Daubentonia madagascariensis*	Aye-aye	Strepsirrhini		PVJZ01004575:1-165882
HCAR2/3	*Eulemur rufifrons*	Bennett's brown lemur	Strepsirrhini		JBFNAD010000021:c5351307-5337816 (Chr21)
HCAR2/3	*Lemur catta*	Ring-tailed lemur	Strepsirrhini	XM_045534921.1	Chr21
HCAR2/3	*Propithecus coquereli*	Coquerel's sifaka	Strepsirrhini		NW_012154285.1:c4068080-4004740
HCAR2/3	*Microcebus murinus*	Gray mouse lemur	Strepsirrhini	XM_012782142.2	Chr21
HCAR2/3	*Mirza coquereli*	Coquerel's mouse lemur	Strepsirrhini		PVHQ01011633:c56256-1
HCAR2/3	*Nycticebus coucang*	Slow loris	Strepsirrhini	XM_053589828.1	Chr4
HCAR2/3	*Otolemur garnettii*	Small-eared galago	Strepsirrhini		NW_003852494.1:3058782-3169648
